# What is causing the rise in food allergy? A narrative review of risk factors for the development of food allergy in infants and children

**DOI:** 10.3389/falgy.2022.1037596

**Published:** 2022-11-24

**Authors:** Nazmul Islam, Derek K. Chu

**Affiliations:** ^1^ Department of Health Research Methods, Evidence and Impact, McMaster University, Hamilton, Ontario, ON, Canada; ^2^ Department of Medicine, McMaster University, Hamilton, Ontario, ON, Canada

**Keywords:** food allergy, risk factors, infants and children, developmental origins and life-course theories, IgE mediated food alergy

## Introduction

Food allergies affect 10% of children globally (1) and are increasingly more common. Anaphylaxis is the most severe form of an allergic reaction and, with foods being the most common trigger, is increasing in incidence in parallel (2). Since there are currently limited treatment options for food allergies, which are frequently lifelong conditions, prevention strategies are needed. Despite the widespread problem and numerous observational studies exploring the allergy epidemic, clear explanations for it, however, remain elusive.

Clinicians, patients and caregivers, and policy makers need to know who is at high risk for developing disease. Terms such as “high risk” should also inform research and the development of evidence-based recommendations for preventing food allergies, but no commonly understood definition exists. Therefore, clinicians, parents, and children are left without the best information to identify children at high risk or potentially modifiable factors that could critically influence food allergy – and healthy child development – trajectories. Knowing the best risk-related evidence could also help clinicians and researchers identify individuals most likely to develop food allergy, enhancing the feasibility and impact of studies.

### Current understanding of risk factors for the development of food allergy in children

Several risk factors have been suggested to contribute to food allergy. However, there is a lack of robust evidence addressing the scope, magnitude of effect, and modifiability of factors that promote or protect against its development. We next summarize some exemplars.

To understand genetic factors that might influence the risk of developing food allergy, a genome-wide association study evaluated major histocompatibility II genes in 2,759 United States (US) participants of European ancestry from the Chicago Food Allergy Study with food allergy (defined as a history of an allergic reaction after ingestion of peanut, egg, or milk and food sensitization to the same food [food-specific immunoglobulin E (IgE) ≥ 0.10 kU/L and/or skin prick test mean wheal diameter ≥3 mm]) (3). They reported HLA-DR (odds ratio [OR], 1.7 [95% CI: 1.4–2.1) and HLA-DQ [OR: 1.7 (95% CI: 1.4–2.1)] to be associated with peanut allergy. Using 106 cases and 76 controls, the same team conducted an epigenome-wide association study using the same data addressing cow's milk allergy. They found altered DNA methylation in genes involved in Th1 and Th2 pathways (*il1rl1, il5ra, stat4, il4,* and *ccl18*), among others to be associated with disease (4). With food allergy being associated with Th2 dominance (5–7), the relevance of the Th1 pathway epigenetic changes is unclear. But one limitation of the study was that they did not report any relative or absolute measures and only reported summary estimates.

More recently, a systematic review (8) assessed genetic determinants of pediatric food allergy. They included 32 studies, with five of them being genome-wide association studies and the rest being candidate gene studies. Twenty-two of the studies involved a predominantly Caucasian population, with the remaining ten involving Asian-specific populations or people of unknown ethnicity. Furthermore, the sample sizes in the included studies varied, with the smallest study having 30 food allergy cases and 35 nonallergic controls, and the largest study being a genome wide association study with 2,197 European patients (671 with food allergy, 144 non-allergic non-sensitized controls and 1,382 European controls of uncertain phenotype about their food allergy status). They mentioned that *flg, hla, il10, il13* were associated with food allergy. Still, the authors made the judgment based on only descriptive results and qualitative judgment, and they did not provide any pooled estimates. To summarize, genetic and epigenetic factors may be associated with food allergy, but the evidence is sparse and their applicability to inform decision making has not reached clinical practice.

In terms of family history, a study used the Chicago Family Cohort Food Allergy database to assess the likelihood that siblings of a person with a food allergy will also develop food sensitization and allergies (9). They defined food allergy status of the children based on the data collected from questionnaire-based interviews with each parent performed by the study staff, results of allergen specific IgE measurements (≥0.35 kUA/L considered as children having food allergy) and skin prick testing (mean wheal diameter >3 mm). They defined clinical reaction if children had a history of reaction within 2 h of ingestion and skin or blood sensitization (IgE ≥ 0.35 kUA/L). Of the 1,120 children meeting their case definition of food allergies and at least one sibling, 67% of the siblings had sensitization, and 14% had clinical reactions. However, their adjusted multinomial regression model showed no clear association for food allergy among siblings (relative risk reduction [RRR], 1.27 [95% CI: 0.94–1.60). On the other hand, another study assessed the impact of a family history of allergic disease on the risk of developing food allergy in the first year by using the HealthNuts population-based cohort (10). They defined food allergy if the children had positive oral food challenge (hives, vomiting, angioedema or anaphylaxis) within 2 h of a dose of the challenge food (egg, peanut or sesame). After multivariable logistic regression, they found that having one immediate family member with a history of any allergic disease [adjusted OR: 1.4 (95% CI: 1.1–1.7)] and two or more allergic family members [adjusted OR: 1.8 (95% CI: 1.5–2.3)] are associated with food allergy in children when compared to those with no family history of allergic disease. These studies show that the limited data addressing genetic factors suggests they could play a minor role in promoting food allergy.

The National Academies of Sciences, Engineering, and Medicine (NAS) described other factors, including non-genetic factors (often environmental), in their 2017 systematic review (11). For example, they concluded weak but consistent evidence that a compromised skin barrier plays a role in sensitization as a precursor to food allergy. The greater likelihood of food allergy in people with mutations in filaggrin, a protein involved in maintaining the skin barrier, favoured the concept (OR: 31 [95% CI: 3 to >100) (12). The result used to support this concept, however, is unreliable and at risk of sparse data bias because of having an estimate far away from the null with extreme confidence intervals (13). A number of recent randomized trials, however, have attempted to improve skin barrier function early in life with the aim of preventing food allergy and they failed to do so (14–16). The relationship between skin barrier impairment and risk for developing food allergy remains to be clarified.

Regarding environmental exposures, a recent review (17) summarized the evidence on risk factors for food allergy with a focus on the outdoor physical environment. They reported that vitamin D insufficiency (<50 nmol/L) (18) at age 12 months [adjusted OR: 11.51 (95% CI: 2.01–65.79)], air pollution- PM_2.5_ (19) [adjusted OR: 1.75 (95% CI: 1.23–2.47)], environmental greenness (20) [adjusted OR: 1.59 (95% CI: 1.05–2.42)], and pollen exposure (21) [adjusted OR: 1.21 (95% CI: 1.01–1.44)] are associated with higher risk in the development of food allergy. They also reported that children reared on farms and near animals [adjusted OR: 0.6 (95% CI: 0.5–0.8)] and children with older siblings and those who attend childcare by six months of age [adjusted OR: 0.5 (95% CI: 0.3–0.8)] are associated with lower risk in the development of food allergy (22). However, we cannot rely entirely on these results as they have a few limitations, i.e., selection bias and high loss to follow-up, which may weaken inferences. Future robust studies are required to address causality.

In summary, evidence to date is conflicting or insufficient as few studies have examined these environmental factors with objective measures of IgE-mediated food allergy. A future systematic and comprehensive research needs to consider the interrelationships between multiple environmental factors.

In terms of modifiable exposures, risks associated with childhood vaccination have been raised, and one argument is that switching to acellular pertussis may have skewed immune responses toward food allergies (23). A cohort of 819 children who received the cellular or acellular vaccine at roughly the same time based on availability found no clear changes in food allergy (adjusted risk ratio [RR], 0.70 [95% CI: 0.26–1.84]) (24).

Another modifiable exposure could be food allergen in the home environment. In a study of infants participating in a study by the United States Consortium for Food Allergy Research (CoFAR) assessed whether environmental peanut exposure is a risk for food allergy or not. They defined food allergy if children had serum IgE ≥ 5 kUA/mL. They found that the amount of peanut in the infants' house dust was associated with an increased risk of developing peanut allergy [OR: 2.10 (95% CI: 1.20–3.67)] (25). Systematic evaluation is required to establish whether this association is reliable.

These are just a few examples that the National Academies and others have identified as possible risk factors. Other examples include inherent risks, such as male sex, genetics (HLA, and specific genes as described above) (26, 27), and potentially modifiable risk factors, such as improved hygiene, the microbiome (28, 29), vitamin D deficiency (18), reduced intake of omega-3-polyunsaturated fatty acids (30), lower consumption of antioxidants (31), increased use of antacids (32), obesity (33), and the timing and method of ingestion of foods (34). Credible evidence proving whether these risk factors are genuinely causal or not, and precise estimates of effect are required in order for them to be actionable. Further, guidance addressing how to apply these numerous potential associations, and deciphering which are spurious or trustworthy, to improve health outcomes is needed.

### Changing recommendations on the timing of food introduction as an intervenable risk factor for the development of food allergy in children

The primary intervenable risk factor identified to date is avoiding common allergens early in life. There is a hypothesis that changing recommendations about food allergy over time is responsible for the higher incidence of food allergy.

In the year 2000, the American Academy of Pediatrics (AAP) committee on nutrition recommended that “breastfeeding mothers should continue breastfeeding for the first year of life or longer: for infants at risk for developing food allergy, hypoallergenic formulas can be used to supplement breastfeeding, and mothers should eliminate peanuts and tree nuts [e.g., almonds, walnuts, etc. (sic)] and consider eliminating eggs, cow's milk and fish from their diets while nursing.” Solid foods should not be introduced into the diet of infants (defined by elevated levels of cord blood IgE and serum IgE in infancy and an atopic family history) until 6 months of age, with dairy products delayed until 1 year, eggs until 2 years, and peanuts, nuts, and fish until 3 years of age (35). Soon after these recommendations, food allergy specialists became concerned about increasing trends in food allergy among young children (36). The AAP revised the recommendation in 2008, stating insufficient data to support the delayed introduction of allergenic foods (37). In the next few years, the idea of primary prevention and using an early introduction to help prevent food allergy came to the front with the publication of the LEAP trial (38). In this trial, they randomly assigned 530 infants with severe eczema, egg allergy, or both between 2006 and 2009 to consume or avoid peanuts until 60 months of age. They reported that introduction of peanuts decreased the frequency (1.9% [*n* = 10] in the consumption group vs. 13.7% [*n* = 73] in the avoidance group; absolute risk difference (ARR), 11.8% [95% CI: 3.4–20.3]) of development of peanut allergy among children. One limitation of the study is that the study excluded 76 children had large wheals after the skin-prick test. The safety and effectiveness of early peanut consumption in that population remain unknown, and the study results' generalizability is questionable as well. In the wake of this report, United States guidelines addressing the prevention of peanut allergy reversed their previous advice to delay the introduction of allergic foods (39, 40). These data show the importance of addressing uncertainty with randomized trials.

Evaluating the impact of the revised guidelines on food allergy incidence, however, is complex. Using the Rochester Epidemiology Project, a retrospective cohort study (41) examined the epidemiology of food allergies among inhabitants of all ages in Olmsted County, Minnesota, over a 10-year period from January 2, 2002, through December 31, 2011, to determine the incidence and temporal trends of food allergies. They defined food allergy based on the following criteria: if participants had IgE ≥ 0.35 kU/L, positive skin prick testing (mean wheal diameter >3 mm), or positive findings on an open food challenge. They found 578 new cases of food allergies and the average annual incidence rate was higher among males compared with females (4.1 [95% CI: 3.6–4.5] vs. 3.0 [95% CI: 2.7–3.4] per 10,000 person-years; 3.6 per 10,000 person-years overall) during the study period. They also reported that between 2002 and 2009, when the AAP released recommendations and withdrew them, there was a significant rise on the incidence of food allergies, followed by stabilization. Between the calendar years 2002–2003 and 2006–2007, the pediatric incidence rate of food allergy increased from 7.0 (95% CI 6.2–8.9) to 13.3 (95% CI 10.9–15.7) per 10,000 before stabilizing at 12.5 and 12.1 in the next two calendar years. Similarly, an Australian study (42) measured the change in population prevalence of peanut allergy in infants after introducing guidelines to feed peanut early in life to try to prevent peanut allergy using two population-based cross-sectional samples (2007–2011 and 2018–2019) of 7,209 infants aged 12 months in Australia. Their main outcome was a change in prevalence estimates over time. To measure the outcome, all infants underwent skin prick tests for peanut and those with positive results underwent oral food challenges. The study revealed no clear decrease in food allergy after publishing guidelines recommending early peanut introduction (prevalence of peanut allergy across the population (2.6% [95% CI: 1.8%–3.4%] in 2018–2019 cohort vs. 3.1% [95% CI: 2.3%–4.1%] in 2007–2011 cohort [difference, −0.5% (95% CI, −1.4% to 0.4%)]. One interpretation of these data hypothesize that no apparent decrease in allergy prevalence could be consistent with the introduction of guidelines averting a continued rise in food allergy. Regardless, these two studies show that translating evidence from randomized trials to population level impact in food allergy is complex and strategies addressing prevention require further study.

### Discussion and insights for future research

Due to advancements in understanding risk factors and possible biomarkers, as well as their integrative, immunological, microbiological, and epigenetic variables, food allergy research is currently in an exciting new phase. The volume of publications is similarly increasing. For instance, The Tolerance induction through Early Feeding to prevent Food Allergy (TEFFA) trial evaluating the effectiveness of introducing egg, cows milk, peanut, and hazelnut *via* a rusk-like feeding powder on food allergy development in the first year of life (43). The Start Eating Early Diet (SEED) trial will experiment to see the role of several common allergens (milk, egg, peanut, cashew, walnut, sesame, soy, and almond) consumed in the first year of life on food allergy outcomes by two years of age (44). From 23 weeks of gestation until the breastfed newborn is four months old, the Australian PrEggNut multicenter trial compares a high peanut and egg diet to a conventional diet, with results for peanut and egg allergy outcomes determined at 12 months of age (45). Furthermore, The Pebbles trial in Australia testing to determine whether applying a ceramide-dominant emollient cream as a skin barrier strategy to high-risk infants from birth is more effective at preventing eczema, food sensitization, and challenge-confirmed food allergy at 12 months of age than using standard skin care (46).

Lack of consensus, however, among patients, clinicians and policymakers regarding food allergy risk factors and recommendations causes uncertainty in clinicians and parents regarding who is at elevated risk. One approach that might prove useful to address potential research waste is to do fewer individual epidemiological studies and instead more robust large-scale studies, including a complete systematic assessment of the current evidence.

Understanding the factors that promote or protect against the increasing allergy outbreak is essential to addressing it. With only one intervention identified to be able to prevent food allergy – early introduction of food allergens – the systematic identification of factors that credibly increase or decrease the risk for food allergy will be crucial to ending the rise in food allergy.
